# Three-dimensional ghost imaging lidar via sparsity constraint

**DOI:** 10.1038/srep26133

**Published:** 2016-05-17

**Authors:** Wenlin Gong, Chengqiang Zhao, Hong Yu, Mingliang Chen, Wendong Xu, Shensheng Han

**Affiliations:** 1 Key Laboratory for Quantum Optics and Center for Cold Atom Physics of CAS, Shanghai Institute of Optics and Fine Mechanics, Chinese Academy of Sciences, Shanghai 201800, China; 2 Research Laboratory for High Density Optical Storage, Shanghai Institute of Optics and Fine Mechanics, Chinese Academy of Sciences, Shanghai 201800, China

## Abstract

Three-dimensional (3D) remote imaging attracts increasing attentions in capturing a target’s characteristics. Although great progress for 3D remote imaging has been made with methods such as scanning imaging lidar and pulsed floodlight-illumination imaging lidar, either the detection range or application mode are limited by present methods. Ghost imaging via sparsity constraint (GISC), enables the reconstruction of a two-dimensional *N*-pixel image from much fewer than *N* measurements. By GISC technique and the depth information of targets captured with time-resolved measurements, we report a 3D GISC lidar system and experimentally show that a 3D scene at about 1.0 km range can be stably reconstructed with global measurements even below the Nyquist limit. Compared with existing 3D optical imaging methods, 3D GISC has the capability of both high efficiency in information extraction and high sensitivity in detection. This approach can be generalized in nonvisible wavebands and applied to other 3D imaging areas.

In daily life, the natural scenes we are facing with, usually are 3D images whereas most modern imaging techniques can only capture the two-dimensional (2D) projection images, losing the depth information of scenes. For example, our familiar Google map and pictures catched by cameras are 2D projection images. Compared with 2D images, 3D imaging is much more advantageous in capturing the characteristics of the targets and 3D remote imaging attracts increasing attentions. Since the laser was invented, some 3D imaging methods like holography and optical coherence tomography have been widely applied in many disciplines, from the life sciences to nanotechnology^1^,^2^. However, these 3D imaging methods are difficult to be extended to 3D remote sensing. A strategy proved to be very successful in 3D remote imaging is to illuminate the target of interest with a narrow pulsed laser and to measure the target’s reflection signals by using a time-resolved technique as done in scanning imaging lidar and pulsed floodlight-illumination imaging lidar^3^,^4^,^5^,^6^. However, it is still difficult for present lidars to achieve high-resolution 3D images of targets in long range. In addition, the reconstruction of 3D images for 3D imaging methods above needs samples at or beyond the Nyquist limit^7^.

Recently, compressive sensing (CS) theory has demonstrated that images, which are sparse in a certain basis, can be stably restored with global measurements below the Nyquist limit^8^,^9^. When CS theory is introduced to the image reconstruction of ghost imaging (GI), sparsity of the target has been taken as a prior constraint in reconstruction and ghost imaging via sparsity constraint (GISC), which is validated by lots of experiments, can obtain a high-resolution 2D image with the measurements below the Nyquist limit even if a single-pixel bucket detector is used to receive the target’s reflected (or transmitted) signals^10^,^11^,^12^,^13^,^14^,^15^,^16^,^17^. The technique quickly arouses enormous interest in remote sensing^16^,^17^,^18^,^19^,^20^, encryption^21^, imaging through scattering media^22^,^23^,^24^, and 3D computational imaging^25^.

For GI, the light fields within the range of axial correlation depth are always spatially correlated^26^, thus all the targets positioned in this range can be clearly imaged even though the detection plane in the reference path is fixed^18^, which provides a necessary condition for 3D GISC. In order to obtain the depth information of the target, similar to lidar, a narrow pulsed laser is used as the source of 3D GISC with pseudo-thermal light and a time-resolved technique is used to measure the target’s reflection signals. Therefore, when the reflection signals from the target are recorded by a time-resolved single-pixel bucket detector, the target’s 3D image can be obtained by a structured image reconstruction method of 3D GISC^27^.

## Results

### Experimental setup

**Figure 1:**
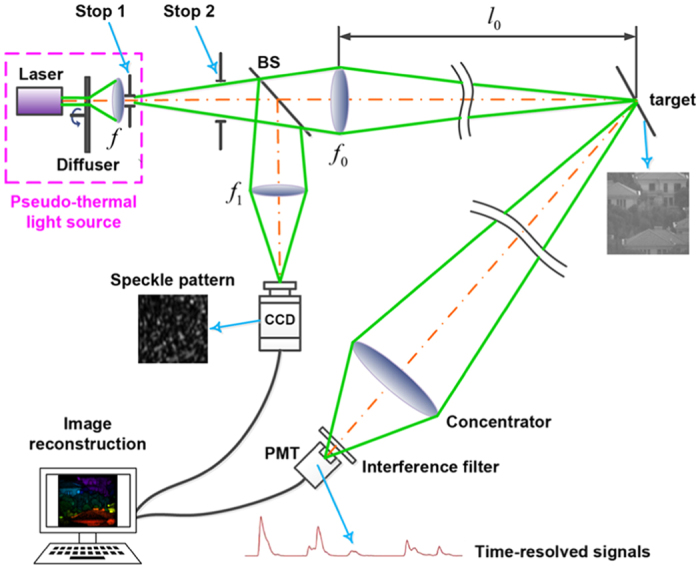
Experimental setup of 3D GISC lidar system with pseudo-thermal light. BS: beam splitter; CCD: charge-coupled device; PMT: photomultiplier tube.

To demonstrate 3D GISC lidar, we constructed the optical setup illustrated in Fig. 1. The pseudo-thermal light source, which consists of a center-wavelength λ = 532 nm solid-state pulsed laser with about 10 ns pulse width at a repetition rate of 500 Hz, a rotating diffuser and a field lens *f*, forms random speckle pattern at stop 1. The laser’s spot diameter illuminating on the rotating diffuser is *D* = 1.65 mm and the distance between the diffuser and the lens *f* = 11.0 mm is 42.5 mm. The diffuser is a ground glass disk controlled by a high-precision motor and the rotating speed of the diffuser is 159 r/min (the maximum linear velocity is 1.0 m/s), which means that the movement amount of the diffuser is 0.01 μm in the sampling time. Therefore, the diffuser can be considered to be static for each sampling and its rotating doesn’t lead to the blur of the speckle pattern. The light emitted from the source goes through the aperture stop 2 and then is divided by a beam splitter (BS) into an object and a reference paths. The distance between the stop 1 and the stop 2 is 255 mm. In the object path, the speckle pattern at stop 1 is imaged onto the target by the objective lens *f*_0_ = 360 mm. The photons reflected from the target are collected by a light concentrator and then pass through an interference filter with 1 nm half-bandwidth into a photomultiplier tube (PMT) connected with a high-speed digitizer of 1 G/s (which is equivalent to a time-resolved single-pixel bucket detector). The bandwidth of the photomultiplier tube is 200 MHz. The transmission aperture of the light concentrator is 140 mm and its focal length is 477 mm. In the reference path, a charge-coupled device (CCD) is placed on the image plane of the speckle pattern at stop 1 and the magnification of imaging system is 1.75×. We have used a Mikrotron CAMMC1362 EoSen mono high-speed CCD camera. The frame frequency of CCD camera was 500 Hz and its exposure time of CCD was set as 5 microseconds in the experiment. In addition, the transverse size of light beam at the objective and reference lens is controlled by the stop 2, which ensures that the entrance pupil is exactly the same for the lens *f*_0_ and *f*_1_. The circular field of view on the target plane for both the transmitting and receiving system is about 34 m/1000 m.

### Measurement and reconstruction

By modulating the laser with a rotating diffuser, we can obtain a series of independent random speckle patterns^12^. In the measurement framework of 3D GISC lidar (see Supplementary Information), the random speckle patterns recorded by the CCD are used to form the sensing matrix **A**^15^,^16^,^17^. Correspondingly, the one-dimensional time-resolved intensity distributions recorded by the PMT are employed to form the measurement data **Y**^15^,^16^,^17^. Exploiting some manifest properties of the target as constraints, both the target’s 3D image and its tomographic image at each time delay (namely at each depth) can be restored by 3D GISC method. In our case, we accept the fact that the target’s images at each depth can be sparsely expressed in a representation basis (or under a suitable basis transform) and the target’s images at different depths have no spatial overlap. In addition, the target’s gray distribution is always real and nonnegative. We have used our proposed structured sparse reconstruction algorithm to recover the target’s 3D images^27^, which can perform the reconstruction in a few minutes on a normal desktop computer.

### Experimental results

**Figure 2:**
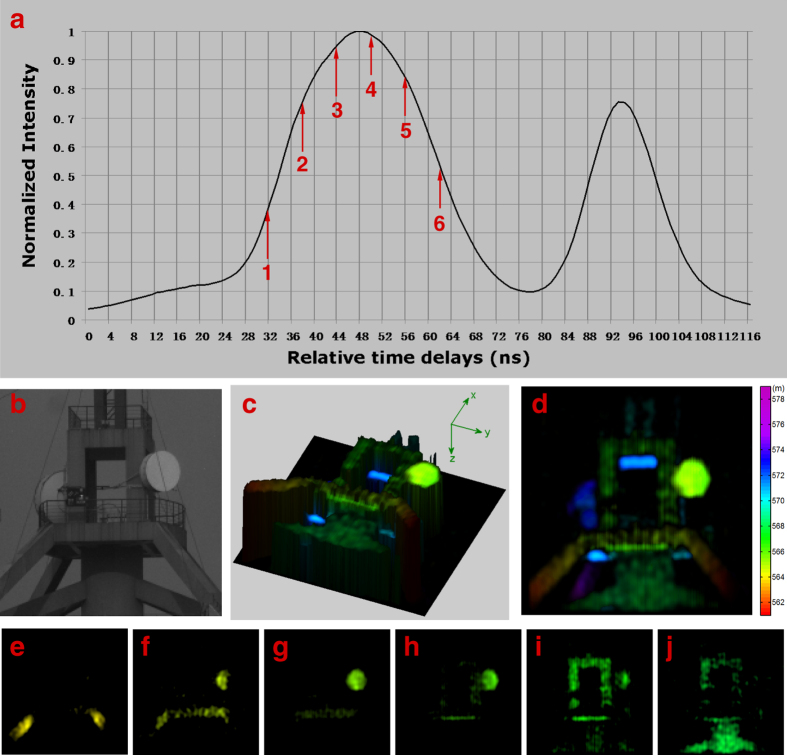
Experimental demonstration results of a tower at about 570 m range, using 6000 measurements. (**a**) The averaged time-resolved signals reflected from the target. (**b**) The original target imaged by a telescope with the receiving aperture 140 mm and the focal length 477 mm. (**c**) The target’s 3D image obtained by 3D GISC lidar. (**d**) x-y projection of the 3D view in (**c**). (**e–j**) The 2D tomographic images of the target reconstructed by 3D GISC method, corresponding to the labeled time delays of Fig. 2(a). The different colors of the images shown in (**c–j**) express different detection distances between the objective lens and the target.

In the experimental demonstration, the transmission aperture of the objective lens *f*_0_ was set as *L* = 3.0 mm by changing the transmission aperture of the stop 2, thus the transverse size of speckle pattern on the target plane (namely the horizontal resolution of the emitting system) was about 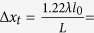 216 mm at a distance of *l*_0_ = 1000 m (see Supplementary Information). The pixel size of CCD was set to be nearly half of the speckle’s full width at half-maximum on the CCD plane. In this case, the speckle’s transverse size on the CCD plane will be 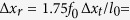 136 μm and we will set the CCD camera’s pixel size as 70 μm × 70 μm. Therefore, the pixel-resolution of speckle patterns recorded by the CCD was about 220 × 220 pixels for a view of field with 34 m/1000 m, which yielded *N* = 12100 resolution cells covering the target (namely the measurement’s Nyquist limit)^13^. In the process of image reconstruction, the target’s 2D image at each depth was represented in two-dimensional discrete cosine transform basis and the measurement number used for reconstructions was 6000 (namely 49.6% of the Nyquist limit). The performance of 3D GISC lidar was first demonstrated by imaging a tower located about *l*_0_ = 570 m away. The target’s projection image, taken with a telescope, is shown in Fig. 2b. Figure 2a presents the measured time-resolved signals reflected from the target, using the time-resolved single-pixel bucket detector in the object path. The time resolution of the measured signals is set as 6 ns in the process of image reconstruction, which implies that the depth resolution of imaging system is 90 cm (see Supplementary Information). Using the structured image reconstruction method of 3D GISC^27^, the tower’s 3D image is displayed in Fig. 2c and its x-y projection image is shown in Fig. 2d. In order to verify the depth resolution and the property of 3D imaging, some of the tower’s tomographic images, labeled for the six time delays in Fig. 2a, are illustrated in Fig. 2e–j, respectively. It is observed that 3D images with the depth resolution of 90 cm can also be obtained for the proposed 3D GISC lidar system even if a laser with 10 ns pulse width is used.

**Figure 3:**
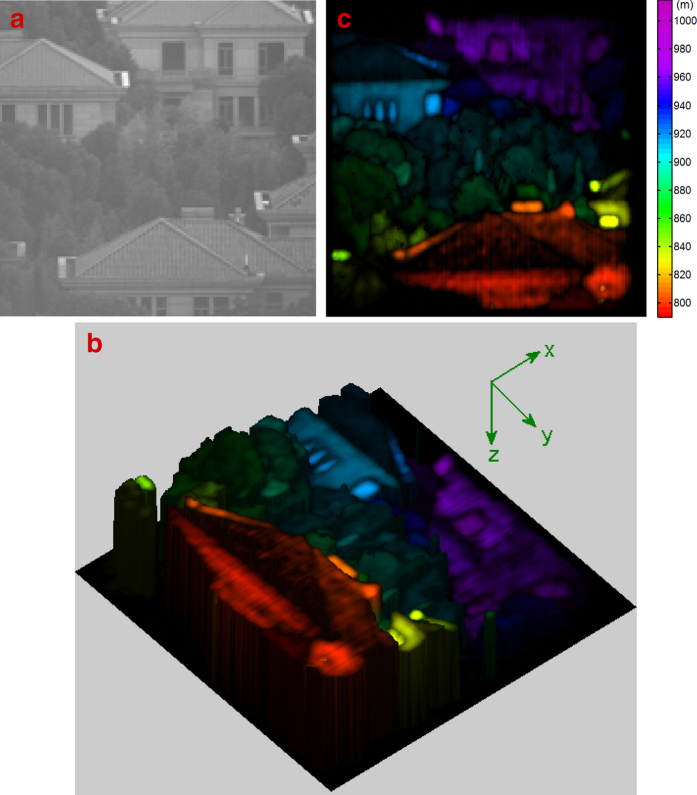
Experimental reconstruction results of imaging a remote sensing scene located about *l*_0_ = 900 m away, using 6000 measurements. (**a**) is the original target captured by the same telescope of Fig. 2. (**b,c**) are the target’s 3D image reconstructed by 3D GISC lidar and its projection image in the x-y plane, respectively.

The experimental demonstration of a 3D remote sensing scene was also shown in Fig. 3, located about *l*_0_ = 900 m away. The picture of the scene captured by the same telescope is displayed in Fig. 3a. Figure 3b,c present the scene’s 3D image achieved by 3D GISC lidar and its projection image in the x-y plane, respectively. As we can see in Fig. 3b,c, the trees, houses and windows located at different depths are clearly visible.

## Discussion

Compared with traditional 3D imaging lidar, 3D GISC lidar has the capability of both high sensitivity in detection and high efficiency in information extraction^8^,^9^,^13^,^14^,^15^,^16^,^17^. Similar to scanning imaging lidar, because all photons collected by the concentrator illuminate the same PMT, 3D GISC lidar has high detection sensitivity for long remote sensing distance and the detection range of 3D GISC lidar is much larger than pulsed floodlight-illumination imaging lidar^3^,^4^. For example, if the effective reflective area of the target for each tomographic image is 50 × 50 pixels, 3D GISC lidar will have about 7 times longer detection range than pulsed floodlight-illumination imaging lidar according to the estimation of radar range equation, even when both the detector’s sensitivity and the system’s transmitting energy for two type of lidars above are the same. Similar to pulsed floodlight-illumination imaging lidar, the laser pulse emitted form the 3D GISC lidar covers the whole detection field and the image can be reconstructed without scanning the target^5^,^6^,^16^. Therefore, in contrast with scanning imaging lidar, 3D GISC lidar can overcome the challenge of the imaging for moving targets with high-speed. What’s more, for a surface 3D scene with *n* × *n* × *n* pixels, its image can be reconstructed by 3D GISC lidar from far less than *n*^2^ pulses. However, for both scanning imaging lidar and pulsed floodlight-illumination imaging lidar, the reconstruction of the surface 3D scene needs samples at or beyond the Nyquist limit^3^,^4^,^5^,^6^,^7^. For example, scanning imaging lidar needs *n*^2^ pulses, while pulsed floodlight-illumination imaging lidar needs a camera with *n* × *n* pixels and high time resolution, which is very difficult to achieve in many wavebands. Although the CCD camera in the reference path is used in our present system, it can be removed by the techniques such as computational ghost imaging^28^ and single-pixel 3D imaging can be achieved, which can overcome the sampling frame frequency of the CCD camera to the imaging speed and has a great application prospect for 3D imaging in the wavelength regions without cameras. In addition, using a much more narrow pulsed laser or chirped-amplitude modulation pulse compression method^29^, the depth resolution of 3D GISC can be dramatically improved. For example, if the pulse width of the laser is 1 picosecond, the depth resolution will be improved to 0.15 mm. Therefore, 3D GISC can also be applied to 3D non-invasive optical imaging in medical diagnosis and a similar 3D microscopy can be proposed to observe the surface morphology of material and biomedical specimens.

## Conclusion

In conclusion, we have developed a 3D GISC lidar system by combining GISC method with time-resolved measurement. We experimentally demonstrate that 3D remote imaging can be achieved with the measurements below the Nyquist limit. This approach of 3D GISC lidar can be generalized to other wavebands and can be applied to other 3D imaging areas, such as 3D non-invasive optical imaging in medical diagnosis and 3D surface microscopy in materials.

## Methods

A series of independent random speckle patterns, obtained by modulating a pulsed laser with a rotating diffuser, were divided by a beam splitter into two paths and then projected onto the target and a charge-coupled device (CCD), respectively. Synchronized by a synchronization controller, the fixed CCD was used to record the 2D gray distribution of speckle patterns and the time-resolved signals reflected from the target were recorded by a time-resolved single-pixel bucket detector. According to the 2D gray distribution of speckle patterns and the corresponding time-resolved reflection signals from the target, both the target’s 3D image and its tomographic image at each depth can be restored by structured sparse image reconstruction method.

## Additional Information

**How to cite this article**: Gong, W. *et al.* Three-dimensional ghost imaging lidar via sparsity constraint. *Sci. Rep.* **6**, 26133; doi: 10.1038/srep26133 (2016).

## Supplementary Material

Supplementary movie 1

Supplementary movie 2

Supplementary Information
