# Supplementary material for: Three-dimensional ghost imaging lidar via sparsity constraint
Source: Sci Rep. 2016 May 17;6:26133. doi: 10.1038/srep26133 (PMC4868975; doi:10.1038/srep26133)
Supplement: Supplementary movie 1 [file srep26133-s1.pdf]

Three-dimensional ghost imaging lidar via sparsity constraint

Supplementary Information

Wenlin Gong, Chengqiang Zhao, Hong Yu, Mingliang Chen,
Wendong Xu, and Shensheng Han

correspondence to: gongwl@siom.ac.cn

1. CS sparse reconstruction

CS sparse reconstruction is a method to finding sparse solutions to under-determined, or ill-conditioned, linear systems of equations[8,9]. The measurement model can be expressed as

$$y = Ax + e = A\Psi\alpha + e. \quad (1)$$

where A is an $M\times N$ sensing matrix ($M<<N$), y (an $M\times1$ vector) is the measurement signals and e (an $M\times1$ vector) denotes the detection noise. x (an $N\times1$ vector) is the original image and can be represented as $x=\Psi\cdot\alpha$ such that α is sparse (namely there are only S non-zero entries in the column vector α, $S<<N$ and Ψ is an $N\times N$ transform matrix). And the convex optimization program of CS sparse reconstruction is[30]

$$x = \Psi\alpha;\ \text{which minimizes: } \frac{1}{2}\|y - Ax\|_2^2 + \tau\|\alpha\|_1. \quad (2)$$

where τ is a nonnegative parameter, $\|V\|_2$ and $\|V\|_1$ represent the Euclidean norm and the ℓ_1-norm of V, respectively.

By now, there are many sparse reconstruction algorithms to solve the convex optimization program described in Eq. (2). For example, gradient projection for sparse reconstruction, iterative shrinkage/thresholding, matching pursuit, ℓ_1-magic, orthogonal matching pursuit, and compressive sampling matching pursuit[30].

2. The measurement framework of 3D GISC lidar

As shown in Supplementary Figure 1, the measurement process of 3D GISC lidar can be expressed as follows in the framework of CS sparse reconstruction:

(1) When the laser emits a pulse, the rotating diffuser will modulate the laser and produce a random speckle pattern. The CCD in the reference path will record the speckle pattern's 2D gray distribution I_i ($m\times n$ pixels, $\forall_i=1\cdots M$) (see Supplementary Figure 1. a_1-a_M) and we reshape each I_i into a row vector ($1\times N$, $N=m\times n$). Correspondingly, the PMT connected with a high-speed digitizer of 1 G/s will record the time-resolved one-dimensional intensity distribution I_i^Q ($1\times Q$ time delays, $\forall_i=1\cdots M$) (see Supplementary

Figure 1. b_1-b_M) because both the time delays and intensities of the light signals reflected form the target's different parts are different.

(2) After M independent modulations, we can obtain M frames of independent 2D gray distributions, then the sensing matrix **A** ($M\times N$) of CS sparse reconstruction can be obtained. Correspondingly, the M independent time-resolved one-dimensional distributions are used to form the measurement data **Y** ($\mathbf{Y}=[y^1, y^2, \ldots, y^q, \ldots, y^Q]$, y^q is an $M\times 1$ vector, $\forall_q = 1 \cdots Q$).

(3) Based on the CS sparse reconstruction model described in Eq. (1), the measurement process of 3D GISC lidar can be expressed as

$$\mathrm{Y} = \mathrm{AX} + \mathrm{E} = \mathrm{A\Psi O} + \mathrm{E}. \tag{3}$$

where **X** ($\mathbf{X} = [x^1, x^2, \ldots, x^q, \ldots, x^Q]$, x^q is an $N\times 1$ vector, $\forall_q = 1 \cdots Q$) denotes the original 3D image and **O** is the sparse representation vector of **X** by Ψ transform. **E** ($\mathbf{E}=[e^1, e^2, \ldots, e^q, \ldots, e^Q]$, e^q is an $M\times 1$ vector, $\forall_q = 1 \cdots Q$) is the detection noise.

3. Image reconstruction of 3D image

Considering the structure property of 3D images in depth direction, the target's 3D image can be reconstructed by solving the following convex optimization program[27]

$$\mathrm{X} = \Psi \mathrm{O};\ \text{which minimizes: } \frac{1}{2}\|\mathrm{Y\text{-}AX}\|_2^2 + \tau\|\mathrm{O}\|_1 + \tau_c \mu(\mathrm{X}). \tag{4}$$

where $\mu(\mathrm{X})$ denotes the mutual coherence function described by Eq. (7) of Ref. (27) and τ_c is a nonnegative parameter determined by the coherence between the tomographic images of neighbor depths.

Based on the image reconstruction algorithm of 3D GISC lidar described above, the animation of rotating the tower and the scene demonstrated in the manuscript are provided by the files entitled *3D-tower.AVI* and *3D-scene.AVI*, respectively.

4. The transverse resolution of the lidar system and the depth resolution

4.1. The design of the system's spatial transverse resolution

In accordance to the principle of ghost imaging, the system's transverse resolution is determined by the transverse size of speckle pattern on the object plane, which is given

by the Van Cittert Zernike theorem, namely $\Delta x = \frac{\lambda z}{w}$, where λ is the wavelength of the source, z is the axial distance and w is the beam waist[11,12,18,27]. For the setup of proposed 3D GISC lidar, in order to build the correlation between two paths, the speckle pattern at stop 1 is imaged onto the target by the objective lens f_0=360 mm and the CCD camera by the reference lens f_1, respectively. What's more, the transverse size of light beam at the objective and reference lens is controlled by the stop 2 shown in Fig. 1, which ensures that the entrance pupil is exactly the same for the lens f_0 and f_1. Therefore, when the transmission aperture of the stop 2 is large enough, the transverse size of speckle pattern on the target plane and the CCD camera are $\Delta x_t = M_t \Delta x$ and $\Delta x_r = M_r \Delta x$, respectively, where M_t and M_r are the magnification of imaging system in the test and reference paths. If the transmission aperture of the stop 2 is too small such that the transmission aperture of the objective lens f_0 and the reference lens f_1 are restricted, then the transverse size of speckle pattern on the target plane and the CCD camera will be $\Delta x_t = \frac{1.22\lambda l_0}{L_t}$ and $\Delta x_r = M_r \frac{\Delta x_t}{M_t}$. For the experimental demonstration, we have chosen the second case. Therefore, when the transverse size of speckle pattern on the target plane is $\Delta x_t = \frac{1.22\lambda l_0}{L_t}$, the transverse size of speckle pattern on the CCD camera will be $\Delta x_r = M_r \frac{\Delta x_t}{M_t} = \frac{M_r f_0 \Delta x_t}{l_0}$.

4.2. The depth resolution for 3D GISC lidar

For 3D GISC lidar, similar to the Rayleigh criterion of spatial transverse resolution, when the difference of the photon's fight time from two objects is smaller than the laser's pulse width, the reflected signal, like the curve shown in Fig.2a, can not be resolved, which will cause the phenomenon that the information of two objects appear in the same tomographic image. However, the relative intensities of the two objects' tomographic images are different in different depths. In the process of 3D image reconstruction, except for exploiting the general assumption of the image's sparsity, we have also used the priori knowledge that the target's images at different depths have no spatial overlap for a

surface 3D images, thus the time delay with the largest intensity for each fixed space position (x,y) will be the distance of the target's corresponding space position range from the lidar system. Based on the technique of image reconstruction, the depth resolution of 3D GISC lidar will be higher than that determined by the laser's pulse width.

References

30. Figueiredo, M. A., Nowak, T. R. D. & Wright, S. J. Gradient Projection for Sparse Reconstruction: Application to Compressed Sensing and Other Inverse Problems. *IEEE J. Sel. Top. in Sig. Proc.* **1**, 586-597 (2007).

Supplementary Figures

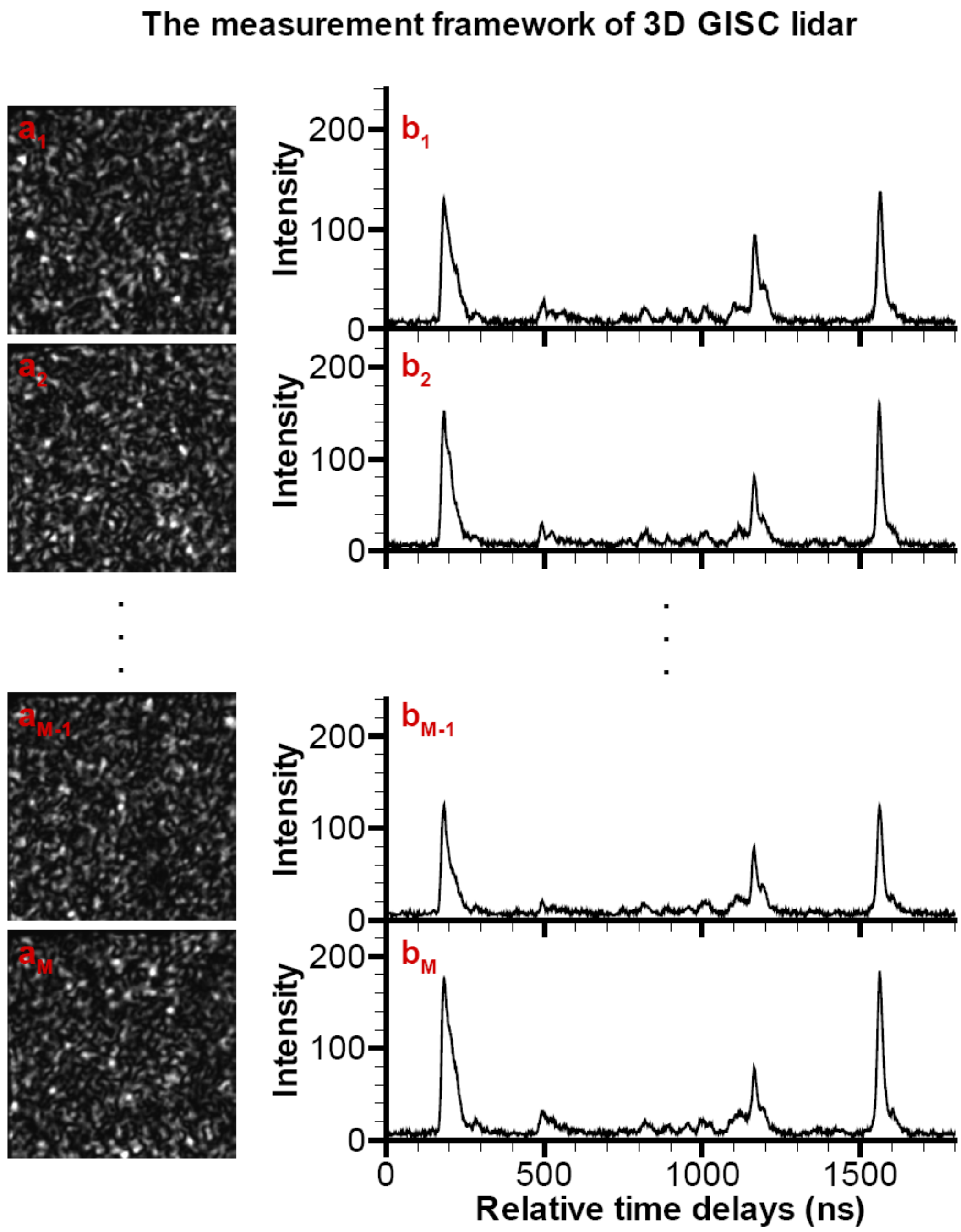

Supplementary Figure 1: The measurement framework of 3D GISC lidar system with pseudo-thermal light. a_1-a_M are the speckle pattern's 2D gray distributions recorded by the reference CCD in different modulations. b_1-b_M are the corresponding time-resolved one-dimensional intensity distribution recorded by the time-resolved single-pixel bucket detector in the object path.

Supplementary captions of Movies

3D-tower.AVI

Supplementary Movie 1. The animation of rotating the tower shown in Fig. 2.

3D-scene.AVI

Supplementary Movie 2. The animation of rotating the scene shown in Fig. 3.
